# New *Saussurea* (Asteraceae) species from Bogeda Mountain, eastern Tianshan, China, and inference of its evolutionary history and medical usage

**DOI:** 10.1371/journal.pone.0199416

**Published:** 2018-07-18

**Authors:** Jie Chen, Yu-Jin Wang

**Affiliations:** State Key Laboratory of Grassland Agro-Ecosystem, School of Life Sciences, Lanzhou University, Lanzhou, PR China; Brigham Young University, UNITED STATES

## Abstract

In this study, ***Saussurea bogedaensis* Yu-J. Wang & Jie Chen**, a new species from Bogeda Mountain in the eastern part of the Tianshan Mountains, is described and discussed based on evidence in terms of both morphological and genetic data. *S*. *bogedaensis* is morphologically similar to *S*. *involucrata*, which is distributed in the western part of the Tianshan Mountains, and it is well known because of its beauty, rarity, and medicinal value. The new species is also similar to *S*. *orgaadayi*, which is distributed in the nearby Altai Mountains. Our genetic data support the close relationships among these three species. According to their allopathic distributions, we suggest that these three species are derived from the same ancestor but that they differentiated after reaching their current range. In addition, we propose that the new species might serve as an alternative to *S*. *involucrata* in medicine due to their very high similarity. However, this species appears to be rare because we only found six mature individuals in the field despite extensive investigations.

## Introduction

*Saussurea involucrata* is well known because of its beauty, rarity, and medicinal value in China. Its Chinese name, i.e., “snow lotus,” refers to its similar appearance to a lotus, which is a well-known ornamental plant. This species is usually found on mountains covered with snow all year around, which enhances its beauty and explains its associations with many mysterious legends. This species has been used for a long time as a traditional Chinese medicine (TCM) to treat a wide spectrum of disorders such as rheumatoid arthritis, tumor diseases, and high-altitude diseases [1–4]. TCM has been modernized and analyses have isolated and identified more than 70 compounds in *S*. *involucrata* [5,6]. In addition, this species has recently been selected as a cold-resistance model in order to exploit its genetic resources [7–9]. Partly due to its over-exploitation, *S*. *involucrata* is currently endangered and included in the list of national second-class protected plants in China [10,11], although a few methods for in vitro propagation have been reported [12–15].

**Fig 1:**
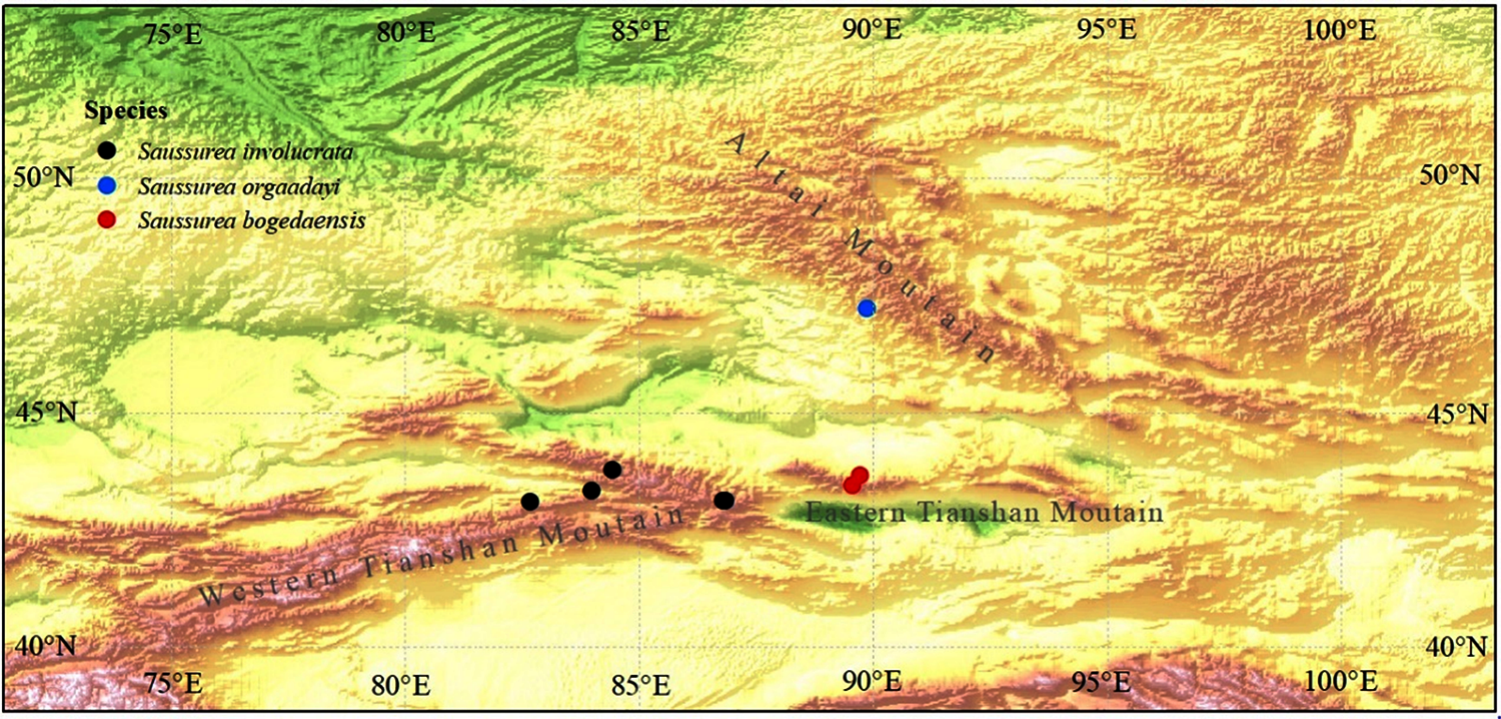
Map showing the locations visited to obtain samples of *Saussurea bogedaensis*, *S*. *orgaadayi*, and *S*. *involucrata*.

In contrast to the public popularity and significant medical value of *S*. *involucrata*, its taxonomic status has received little attention. It was considered to be widespread in the Tianshan Mountains and the nearby Altai Mountains, but recently the population in the Altai Mountains was ascribed to a new species called *S*. *orgaadayi* [16]. This species was generally recognized as *S*. *involucrata* in the local medicine market, but it can be differentiated from *S*. *involucrata* based on a number of morphological features such as the phyllary and involucre [16–19]. This unexpected taxonomic finding suggests that all of the populations cannot be treated as a single species throughout the Tianshan Mountains, which stretch 4000 km from the west to east with a width of up to 150 km in [20]. The Tianshan Mountains are divided into two parts around the Chaiwopu basin of Urumqi at a longitude of about 88° [20,21], where the western part is called Western Tianshan and the eastern part is called Bogeda Mountain (Fig 1).

To explore the possible differentiation of *S*. *involucrata* along the Tianshan Mountains from east to west, we conducted a field investigation in 2013 and found considerable differences according to the geographical regions. However, the eastern population is very small and we only found two individuals in a restricted area immediately below a peak that is covered with snow throughout the year. Thus, we made a second trip via another road in 2016. Once again, we failed to find a large population and encountered only four mature individuals and no more than 50 immature individuals.

Based on the results obtained in the present study, we propose to name the population found in the eastern part of the Tianshan Mountains as a new species called *Saussurea bogedaensis* Yu-J. Wang & Jie Chen. We obtained photographs in the field and determined the major differences compared with *S*. *involucrata* and *S*. *orgaadayi*. In order to determine its taxonomic status, we analyzed the genetic diversity based on the nuclear internal transcribed spacer (ITS) and three chloroplast (cp) loci for the new species and 18 other representative species of subg. *Amphilaena*, which includes *S*. *involucrata* and *S*. *orgaadayi* [17].

## Materials and methods

### Ethics statement

All the collecting locations are not in any natural conservation area and no specific permissions were required for these locations. One protected species (*Saussurea involucrata*) was collected with introduction letters of School of Life Sciences Lanzhou University and permission from Urumqi Forestry Bureau. The individual in this manuscript has given written informed consent (as outlined in PLOS consent form) to publish these case details.

### Taxon sampling for molecular phylogeny reconstruction

In total, 44 accessions were sampled, including eight accessions of the new species (*S*. *bogedaensis*) from two populations on Bogeda Mountain, four accessions of *S*. *orgaadayi* from one population in the Altai Mountains, 15 accessions of *S*. *involucrata* from five populations in the Tianshan Mountains, 16 accessions representing the remaining species in subg. *Amphilaena*, and one accession comprising *Jurinea multiflora* as an outgroup. Fresh leaves were dried immediately after sampling with silica gel for DNA extraction. Voucher specimens were deposited in the herbarium at Lanzhou University (LZU). The detailed geographical locations of each sampled population are shown in Fig 1 and Table 1.

**Table 1:** Origins of materials (all these samples are from China) and GenBank accession numbers (ITS, *mat*K, *psb*A-*trn*H, and *trn*K).

Taxon	Origin	North latitude (°)	East longitude (°)		Altitude (m)	GenBank accession no.
*S*. *bracteata*	Yushu, Qinghai; WYJ201607043	35.05681	93.01222	4644	MF680674, MF680714, MF680754, MF680794	
*S*. *erubescens*	Luqu, Gansu; SN110814017	34.59414	102.48834	3421	MF680675, MF680715, MF680755, MF680795	
*S*. *wettsteiniana*	Mianning, Sichuan; WYJ201607402	29.00106	102.14985	3381	MF680688, MF680717, MF680757, MF680797	
*S*. *globosa*	Kangding, Sichuan; W201209158	30.05502	101.95973	3992	MF680676, MF680716, MF680756, MF680796	
*S*. *uniflora*	Cuona, Xizang; WYJ201607254	27.76583	91.90194	4138	MF680685, MF680718, MF680758, MF680798	
*S*. *nigrescens*	Menyuan, Qinghai; LJQ-QLS-2008-065	37.40971	101.67202	2800	MF680679, MF680719, MF680759, MF680799	
*S*. *veitchiana*	Shenlongjia, Hubei; WYJ201507160	31.43997	110.30714	3098	MF680686, MF680720, MF680760, MF680800	
*S*. *iodostegia*	Datong; Shanxi; WYJ201507117	39.05578	113.65927	2514	MF680677, MF680721, MF680761, MF680801	
*S*. *pubifolia*	Jiacha, Xizang; WYJ201607272	29.03175	92.35724	4796	MF680683, MF680722, MF680762, MF680802	
*S*. *velutina*	Xiaojin, Sichuan; WYJ201209124	30.99441	102.82915	4000	MF680687, MF680723, MF680763, MF680803	
*S*. *polycolea*	Linzhi, China; LJQ07257	29.36866	94.39168	4680	MF680682, MF680724, MF680764, MF680804	
*S*. *tangutica*	Gansu; WYJ201607013	38.60685	99.48221	4096	MF680684, MF680725, MF680765, MF680805	
*S*. *luae*	Linzhi, Xizang; WYJ201607286	29.59022	94.59631	4121	MF680678, MF680726, MF680766, MF680806	
*S*. *phaeantha*	Gansu; WYJ201607014	38.60685	99.48221	4096	MF680681, MF680727, MF680767, MF680807	
*S*. *obvallata*	Cuona, Xizang; WYJ201607242	27.92057	91.84863	3970	MF680680, MF680728, MF680768, MF680808	
*S*. *muliensis*	Unpublished data in GenBank	---	---	---	AB254665, ---, ---, ---	
*S*. *involucrata*	Urumqi, Xinjiang; WYJ201607025 (163)	43.10847	86.84220	3564	MF680689, MF680741, MF680781, MF680821	
*S*. *involucrata*	Urumqi, Xinjiang; WYJ201607025 (165)	43.10847	86.84220	3564	MF680690, MF680742, MF680782, MF680822	
*S*. *involucrata*	Urumqi, Xinjiang; WYJ201308203 (41)	43.11985	86.82125	3768	MF680691, MF680744, MF680784, MF680824	
*S*. *involucrata*	Urumqi, Xinjiang; WYJ201308203 (42)	43.11985	86.82125	3768	MF680692, ---,---, ---	
*S*. *involucrata*	Urumqi, Xinjiang; WYJ201308203 (372)	43.11985	86.82125	3768	MF680693, MF680743, MF680783, MF680823	
*S*. *involucrata*	Urumqi, Xinjiang; WYJ201308203 (374)	43.11985	86.82125	3768	MF680694, ---, ---, ---	
*S*. *involucrata*	Tekesi, Xinjiang; WYJ201308184 (24)	43.09915	82.68382	3678	MF680695, MF680738, MF680778, MF680818	
*S*. *involucrata*	Tekesi, Xinjiang; WYJ201308184 (25)	43.09915	82.68382	3678	---, MF680739, MF680779, MF680819	
*S*. *involucrata*	Tekesi, Xinjiang; WYJ201308184 (26)	43.09915	82.68382	3678	MF680696, MF680740, MF680780, MF680820	
*S*. *involucrata*	Dushanzi, Xinjiang; WYJ201308131 (60)	43.77545	84.45615	2684	---, MF680734, MF680774, MF680814	
*S*. *involucrata*	Dushanzi, Xinjiang; WYJ201308131 (61)	43.77545	84.45615	2684	MF680697, MF680733, MF680773, MF680813	
*S*. *involucrata*	Dushanzi, Xinjiang; WYJ201308131 (63)	43.77545	84.45615	2684	MF680698, ---, ---, ---	
*S*. *involucrata*	Xinyuan, Xinjiang; WYJ201308188 (47)	43.33469	84.01032	3543	MF680699, MF680735, MF680775, MF680815	
*S*. *involucrata*	Xinyuan, Xinjiang; WYJ201308188 (48)	43.33469	84.01032	3543	MF680700, MF680736, MF680776, MF680816	
*S*. *involucrata*	Xinyuan, Xinjiang; WYJ201308188 (390)	43.33469	84.01032	3543	MF680701, MF680737, MF680777, MF680817	
*S*. *bogedaensis*	Qitai, Xinjiang; WYJ201607018 (140)	43.45321	89.55213	3471	MF680702, MF680748, MF680788, MF680828	
*S*. *bogedaensis*	Qitai, Xinjiang; WYJ201607018 (166)	43.45321	89.55213	3471	MF680703, MF680745, MF680785, MF680825	
*S*. *bogedaensis*	Qitai, Xinjiang; WYJ201607018 (167)	43.45321	89.55213	3471	MF680704, MF680746, MF680786, MF680826	
*S*. *bogedaensis*	Qitai, Xinjiang; WYJ201607018 (378)	43.45321	89.55213	3471	MF680705, MF680747, MF680787, MF680827	
*S*. *bogedaensis*	Qitai, Xinjiang; WYJ201308006 (38)	43.44370	89.58167	3386	MF680707, MF680751, MF680790, MF680831	
*S*. *bogedaensis*	Qitai, Xinjiang; WYJ201308006 (39)	43.44370	89.58167	3386	MF680708, MF680750, MF680791, MF680830	
*S*. *bogedaensis*	Qitai, Xinjiang; WYJ201308006 (40)	43.44370	89.58167	3386	MF680709, MF680752, MF680792, MF680832	
*S*. *bogedaensis*	Qitai, Xinjiang; WYJ201308006 (309)	43.44370	89.58167	3386	MF680706, MF680749, MF680789, MF680829	
*S*. *orgaadayi*	Altay, Xinjiang; WYJ201308041 (11)	47.21846	89.87999	3541	MF680712, MF680732, MF680772, MF680812	
*S*. *orgaadayi*	Altay, Xinjiang; WYJ201308041 (12)	47.21846	89.87999	3541	MF680713, MF680731, MF680771, MF680811	
*S*. *orgaadayi*	Altay, Xinjiang; WYJ201308041 (360)	47.21846	89.87999	3541	MF680711, MF680730, MF680770, MF680810	
*S*. *orgaadayi*	Altay, Xinjiang; WYJ201308041 (361)	47.2184691	89.87999856	3541	MF680710, MF680729, MF680769, MF680809	
*Jurinea multiflora*	Tuoli, Xinjiang; WYJ201308102	45.73564	83.14712	1753	MF680673, MF680753, MF680793, MF680833	

### Morphological observations

Morphological descriptions were prepared based on examinations of the fresh and pressed specimens. Specimens deposited in E, K, PE, KUN, QTPMB, and LZU were examined to make morphological comparison with similar species, i.e., *S*. *orgaadayi* and *S*. *involucrata*. In order to determine the floral micromorphology, dry florets were boiled in distilled water for 5–10 min and photographed under a stereomicroscope (Olympus MD-90).

### DNA extraction and sequencing

Total DNA was extracted from leaf tissues dried with silica gel or herbarium specimens using the modified CTAB method [22]. Four markers were employed comprising ITS, *trn*K, *mat*K, and *psb*A-*trn*H. The primers [23–26] used for amplification and sequencing are listed in Table 2. PCR was performed as described in our previous study [27]. PCR products were sent to Beijing Genomics Institute (BGI) for commercial sequencing. Sequences were aligned using CLUSTALX v.2.1 [28] with the default settings and adjusted manually with Bioedit v.7.0.5 [29]. All of the sequences were registered in GenBank.

**Table 2:** List of the primers used in this study.

Fragment	Primer 1	Sequence (5′–3′)	Primer 2	Sequence (5′–3′)
ITS	ITS1	TCCTCCGCTTATTGATATGC	ITS4	AGAAGTCGTAACAAGGTTTCCGTAGG
*trn*K	*trn*K(UUU)	TTAAAAGCCGAGTACTCTACC	*rps*16	AAAGTGGGTTTTTATGATCC
*trn*H*-psb*A	*psb*A	GTTATGCATGAACGTAATGCTC	*trn*H	CGCGCATGGTGGATTCACAATCC
*mat*K	*mat*K-XF	TAATTTACGATCAATTCATTC	5r	GTTCTAGCACAAGAAAGTCG

### Data analysis

Three datasets were constructed where one comprised the nuclear ITS sequences, the second contained the concatenated sequences of *psb*A-*trn*H, *mat*K, and *trn*K, and the third of all the sequences after the incongruence length difference test that revealed little incongruence (P > 0.01) between chloroplast and ITS data [30]. MEGA v.4.0 was used to calculate the genetic distances under the Kimura two-parameter model [31]. Phylogenetic analyses were conducted using PAUP v.4.0b10 [32] and MrBayes v.3.2.1 [33]. Maximum parsimony (MP) searches were performed using heuristic search methods with tree bisection reconnection branch swapping and equal weighting for all characters. The analyses were repeated 1,000 times with a random order of sequence addition in order to sample multiple islands of the most parsimonious trees. Bootstrap tests were conducted to evaluate node support using 1,000 replicates with heuristic search settings identical to those for the original search. Bayesian inference (BI) was conducted using the different models selected by Modeltest [34] for each partition. Ten million generations were run to estimate parameters related to sequence evolution and likelihood probabilities using the Markov chain Monte Carlo method. Trees were collected every 1000 generations. Tracer v.1.5 (http://tree.bio.ed.ac.uk/software/tracer/) was used to choose a suitable burn-in period. PAUP* v.4.0b10 [32] was used to calculate a consensus tree and posterior probabilities (PP) from the sampled trees after the burn-in period.

### Nomenclature

The electronic version of this article in Portable Document Format (PDF) in a work with an ISSN or ISBN will represent a published work according to the International Code of Nomenclature for algae, fungi, and plants, and hence the new names contained in the electronic publication of a PLOS ONE article are effectively published under that Code from the electronic edition alone, so there is no longer any need to provide printed copies.

In addition, new names contained in this work have been submitted to IPNI, from where they will be made available to the Global Names Index. The IPNI LSIDs can be resolved and the associated information viewed through any standard web browser by appending the LSID contained in this publication to the prefix http://ipni.org/. The online version of this work is archived and available from the following digital repositories: PubMed Central and LOCKSS.

## Results

### Morphological features

**Fig 2:**
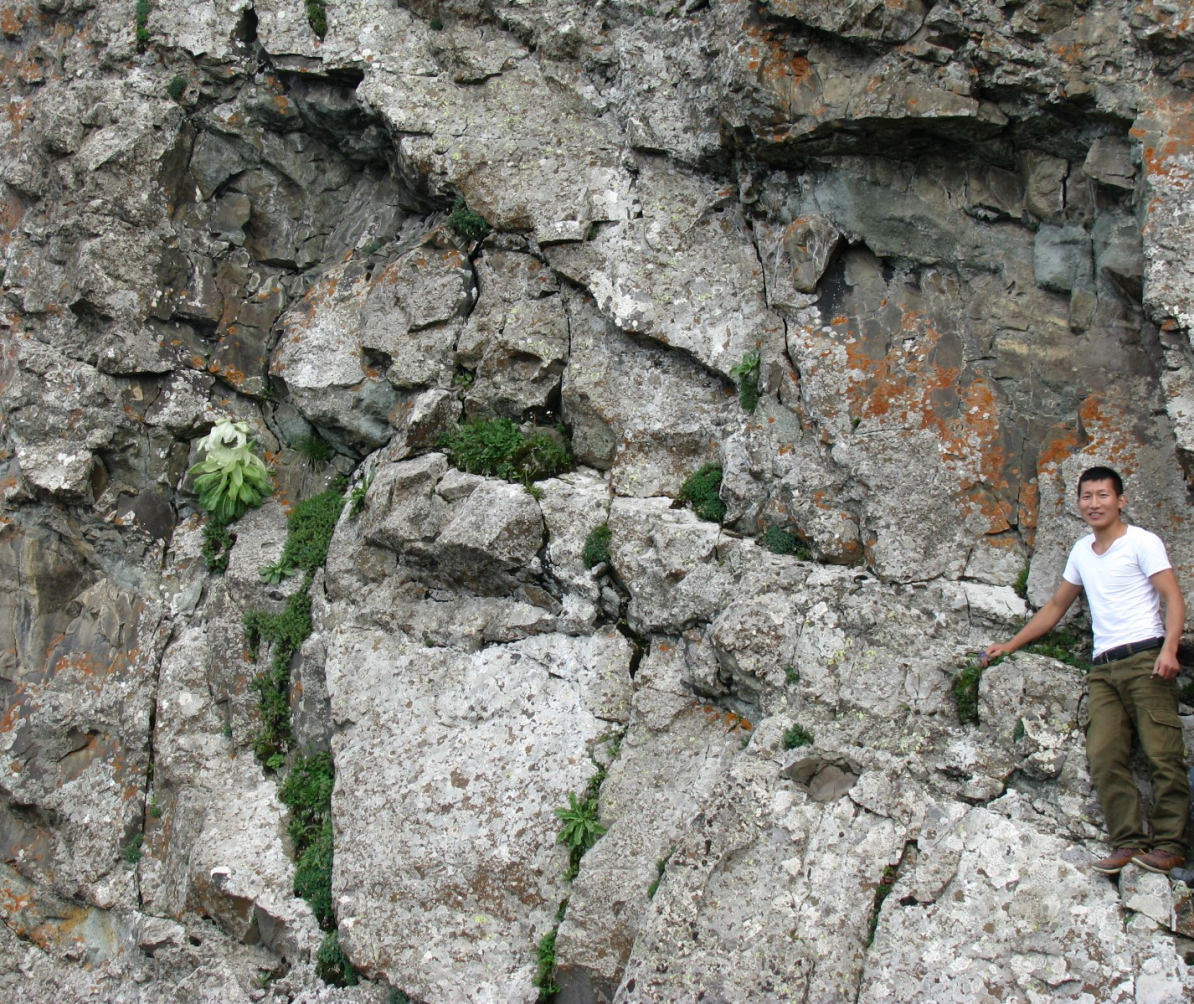
*Saussurea bogedaensis* in the wild.

**Fig 3:**
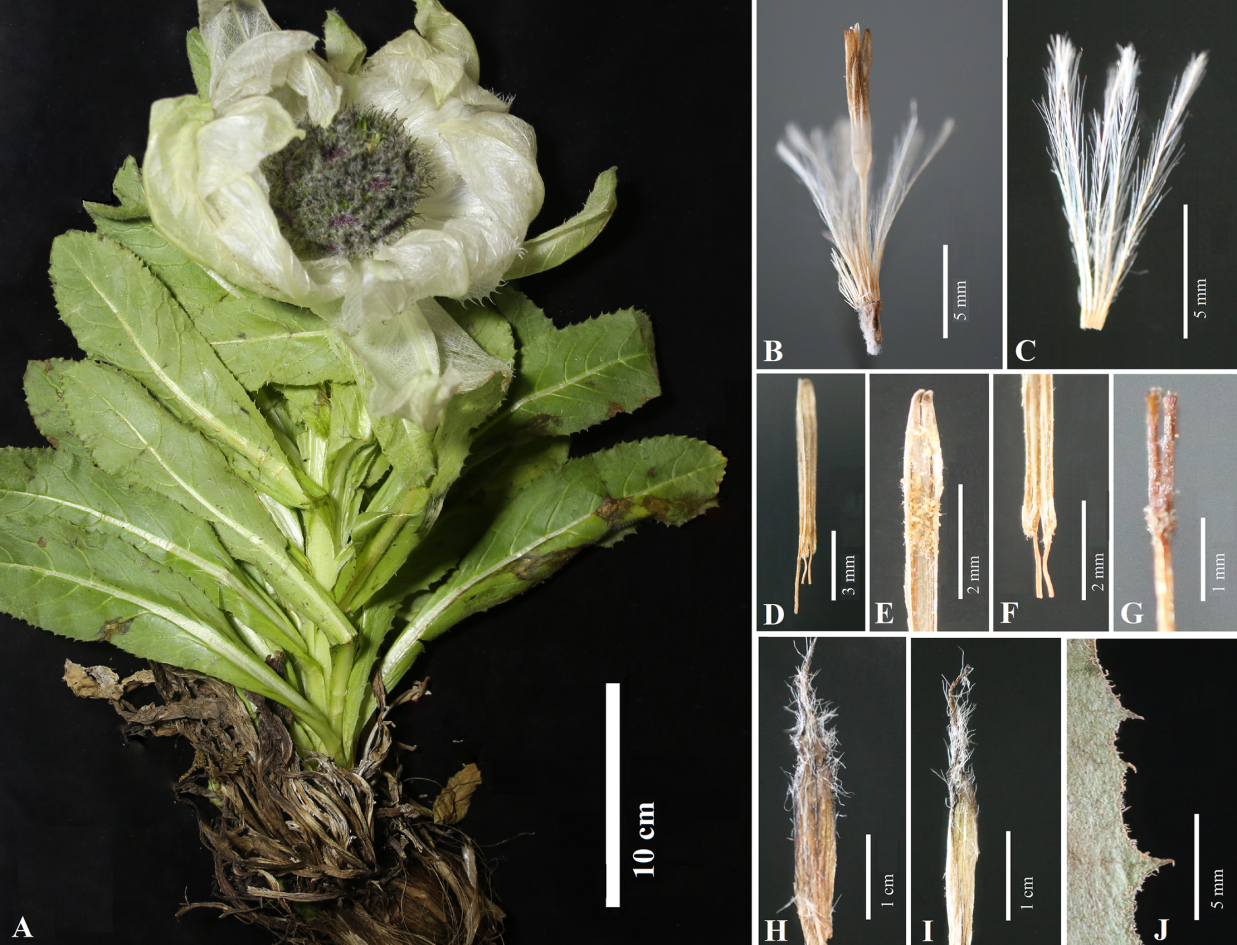
Holotype of *Saussurea bogedaensis (WYJ201607018)*. (A) Living plant; (B) Floret; (C) Inner pappus bristle; (D, E, F) Anthers. (G) Style branches; (H, I) Phyllaries; (J) Stem leaf margin.

**Fig 4:**
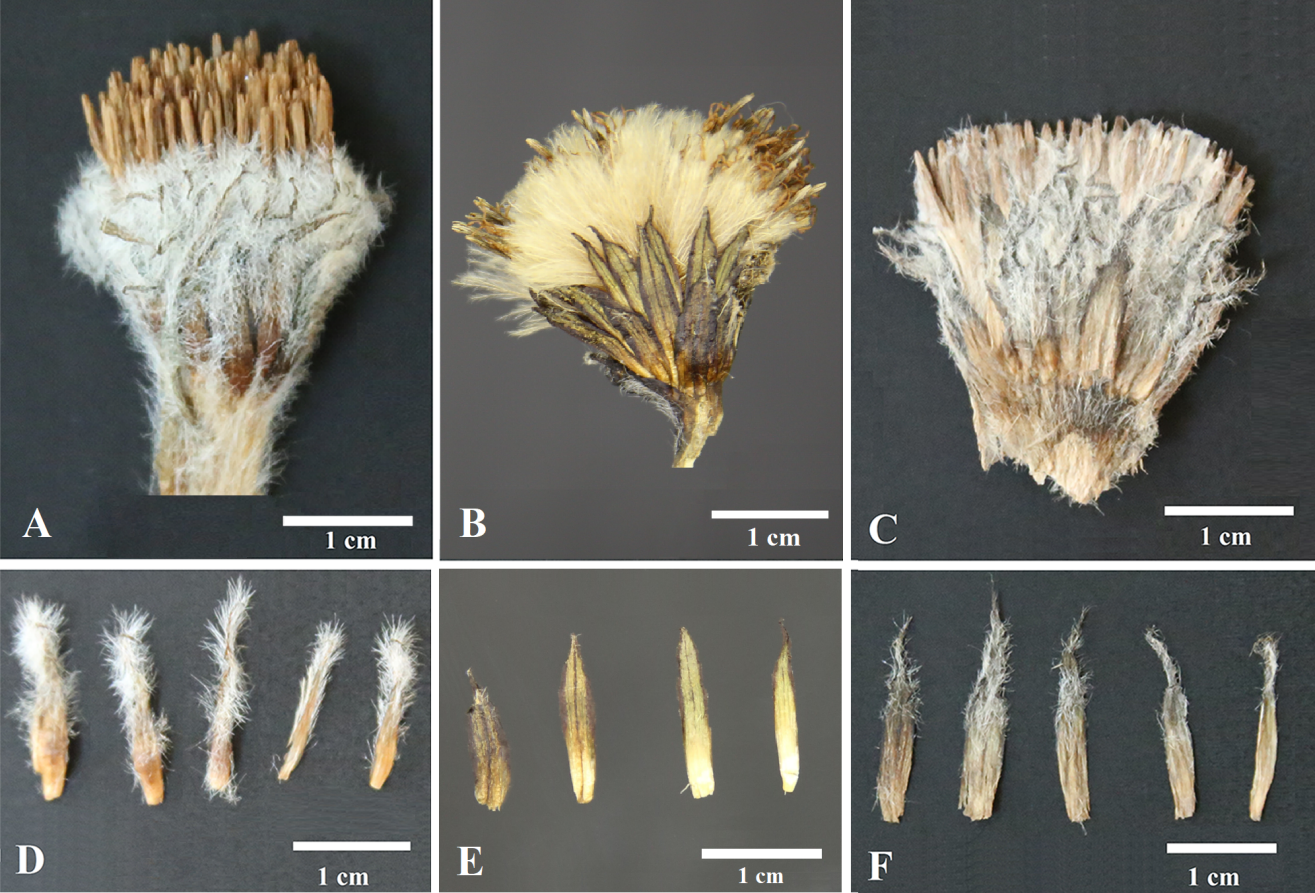
**Comparison of materials from *Saussurea orgaadayi* (A, D), *S*. *involucrata* (B, E), and *S*. *bogedaensis* (C, F).** (A, D) from *WYJ201308041*; (B, E) from *WYJ201607025*; (C, F) from *WYJ201607018*.

Figs 2 and 3 shows photographs of *S*. *bogedaensis*, including the habitat (Fig 2) and close-ups of the florets, pappus, anthers, style branches, phyllaries, and leaf margin (Fig 3). The new species could be differentiated from *S*. *involucrata* or *S*. *orgaadayi* mainly based on the shape of the phyllaries and the indumentum. In the new species, they were acuminate and covered with sericeous-villous in the upper half (Fig 4), whereas they were long, acuminate, and densely pubescent throughout the phyllaries or mostly glabrous in *S*. *involucrata* and *S*. *orgaadayi*. In addition, the three species differed in terms of their leaf, bract, and pappus features, as described in Table 3.

**Table 3:** Comparison of *Saussurea involucrata*, *S*. *orgaadayi*, and *S*. *bogedaensis*.

Features	*S*. *involucrata*	*S*. *orgaadayi*	*S*. *bogedaensis*
Distribution	Western Tianshan Mountains	Altai Mountains	Eastern Tianshan Mountains (Bogeda Mountain)
Petiolar remains of basal leaves	dark brown stripes up to 2–3 mm wide	yellowish brown stripes up to 1 cm wide	dark brown stripes up to 2–3 mm wide
Stem leaves	narrowly ovate, elliptic, or obovate, apex acute, 8–13 × 2–4cm	lanceolate, apex long acuminate 8–17 × 2–5.5 cm,	elliptic, apex obtuse, 15–20 × 3–5 cm
Bracts	ovate-elliptic, apex acute 5.5–12 × 3.5–6.5 cm	triangular-ovate, apex long acuminate 4–12 × 1.5–6.5cm	ovate-elliptic, apex acute 5.5–12 × 3.5–6.5 cm
Capitula number	10–20	20–30	15–30
Involucre	hemispheric	campanulate	campanulate
Phyllary	triangular-ovate, apex acute or obtuse, phyllaries glabrous, rarely sparsely pubescent apically or along midvein	linear-subulate, apex long acuminate, phyllaries densely pubescent throughout	subulate to acuminate, phyllaries densely pubescent middle-upper part
Pappus color	dirty white	straw-colored	dirty white

### Molecular analyses

**Fig 5:**
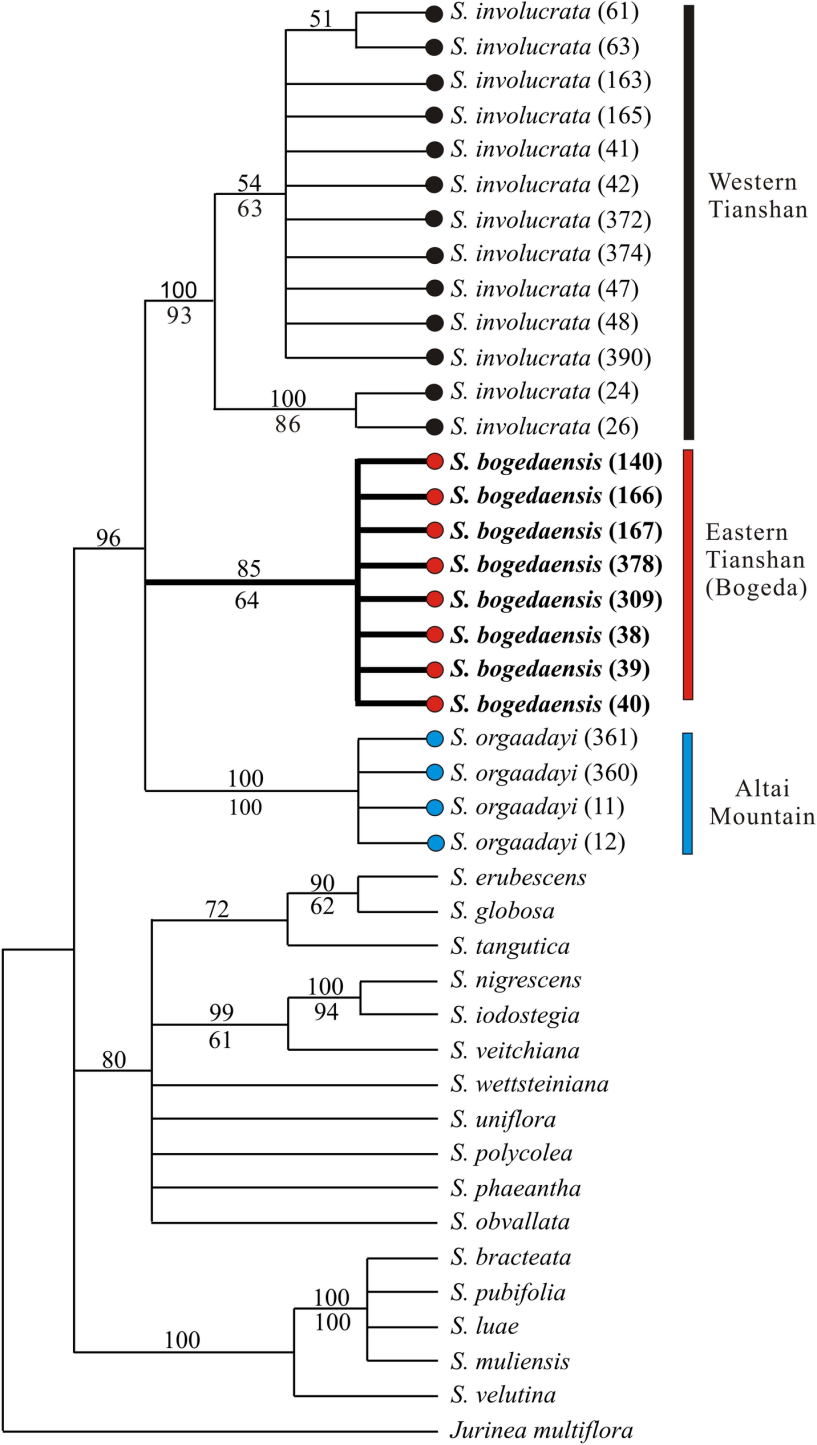
The 50% majority rule consensus tree derived from Bayesian analysis of the nuclear internal transcribed spacer. Posterior probabilities (PPs) and bootstrap percentages (BPs) are indicated above and below the branches, respectively.

The aligned ITS data sets comprised 20 taxa with 607 positions and 69 variable characters, where 33 were parsimony informative when gaps were treated as missing. The mean pairwise distance within subg. *Amphilaena* was 1.4%. Those between *S*. *bogedaensis* and *S*. *involucrata* or *S*. *orgaadayi* were 0.98% or 2.0%, respectively (Table 4). Two approaches (MP and BI) obtained largely congruent tree topologies. The BI tree is shown in Fig 5 where the Bayesian PPs and MP bootstrap percentages (BPs) are denoted above or below the branches, respectively. We analyzed all three species with multiple individuals, i.e., *S*. *bogedaensis* (PP = 85%; BP = 64%), *S*. *involucrata* (PP = 100%; BP = 93%), and *S*. *orgaadayi* (PP = 100%; BP = 100%), and they were found to be monophyletic. Moreover, the three species formed a monophyletic clade (PP = 96%), whereas the other species clustered into two clades.

**Table 4:** Pairwise distances (%) for internal transcribed spacer (lower left) and combined plastid (upper right) sequences from 19 *Saussurea* species.

CP ITS	1	2	3	4	5	6	7	8	9	10	11	12	13	14	15	16	17	18	19
**1**		0.30	0.10	0.40	0.20	0.20	0.30	0.20	0.20	0.30	0.20	0.20	0.20	0.30	0.20	0.20	0.50	0.20	----
**2**	0.98		0.30	0.60	0.40	0.40	0.50	0.40	0.40	0.50	0.40	0.40	0.40	0.50	0.40	0.40	0.70	0.40	----
**3**	2.00	1.37		0.40	0.30	0.30	0.30	0.30	0.20	0.30	0.20	0.20	0.20	0.30	0.20	0.30	0.50	0.20	----
**4**	1.61	0.99	1.71		0.50	0.40	0.60	0.50	0.50	0.50	0.50	0.50	0.50	0.60	0.50	0.40	0.70	0.50	----
**5**	1.79	1.16	1.88	0.50		0.30	0.20	0.10	0.10	0.50	0.10	0.10	0.10	0.20	0.30	0.40	0.70	0.10	----
**6**	1.45	1.17	1.89	0.50	0.66		0.30	0.30	0.20	0.20	0.20	0.20	0.20	0.30	0.30	0.10	0.50	0.20	----
**7**	1.84	1.17	2.24	1.34	1.51	1.52		0.20	0.10	0.50	0.10	0.10	0.10	0.00	0.40	0.50	0.70	0.10	----
**8**	1.57	1.51	2.42	1.51	1.69	1.69	2.05		0.10	0.50	0.10	0.10	0.10	0.20	0.30	0.40	0.70	0.10	----
**9**	2.68	2.00	3.08	2.34	2.52	2.53	0.83	2.90		0.40	0.00	0.00	0.00	0.10	0.30	0.30	0.60	0.00	----
**10**	2.69	2.17	2.91	1.50	1.67	1.67	2.54	2.37	3.56		0.40	0.40	0.40	0.50	0.40	0.10	0.60	0.40	----
**11**	2.15	1.52	2.20	1.17	1.35	1.35	2.05	1.36	2.90	2.03		0.00	0.00	0.10	0.30	0.30	0.60	0.00	----
**12**	2.13	1.50	2.22	0.83	1.00	1.00	1.85	1.35	2.87	1.67	0.50		0.00	0.10	0.30	0.30	0.60	0.00	----
**13**	2.16	1.50	2.56	1.84	2.01	2.01	0.33	2.03	1.16	2.69	2.03	2.01		0.10	0.30	0.30	0.60	0.00	----
**14**	2.63	2.34	3.07	2.00	2.17	2.18	2.19	2.71	3.03	3.20	2.54	2.51	2.51		0.40	0.50	0.70	0.10	----
**15**	2.47	1.84	2.35	1.16	1.33	1.34	1.86	2.38	3.21	2.35	1.69	1.67	2.70	2.86		0.30	0.60	0.30	----
**16**	2.52	2.00	2.18	1.33	1.50	1.50	2.20	2.38	3.38	1.50	1.69	1.50	2.86	3.02	1.84		0.50	0.30	----
**17**	2.36	1.84	2.01	1.16	1.34	1.34	2.20	2.21	3.22	1.33	1.52	1.34	2.70	2.86	1.68	0.16		0.60	----
**18**	1.95	1.33	2.05	0.66	0.66	0.83	1.68	1.69	2.69	1.67	1.18	1.00	2.01	2.34	1.50	1.67	1.50		----
**19**	2.33	1.66	2.74	2.00	2.18	2.18	0.50	2.55	1.33	3.21	2.38	2.52	0.83	2.68	2.87	3.03	2.87	2.18	

1. *S*. *bogedaensis*, 2. *S*. *orgaadayi*, 3. *S*. *involucrata*, 4. *S*. *obvallata*, 5. *S*. *phaeantha*, 6. *S*. *globosa*, 7. *S*. *wettsteiniana*, 8. *S*. *uniflora*, 9. *S*. *polycolea*, 10. *S*. *erubescens*, 11. *S*. *nigrescens*, 12. *S*. *iodostegia*, 13. *S*. *luae*, 14. *S*. *pubifolia*, 15. *S*. *tangutica*, 16. *S*. *muliensis*, 17. *S*. *veitchiana*, 18. *S*. *velutina*, 19. *S*. *bracteata*.

**Fig 6:**
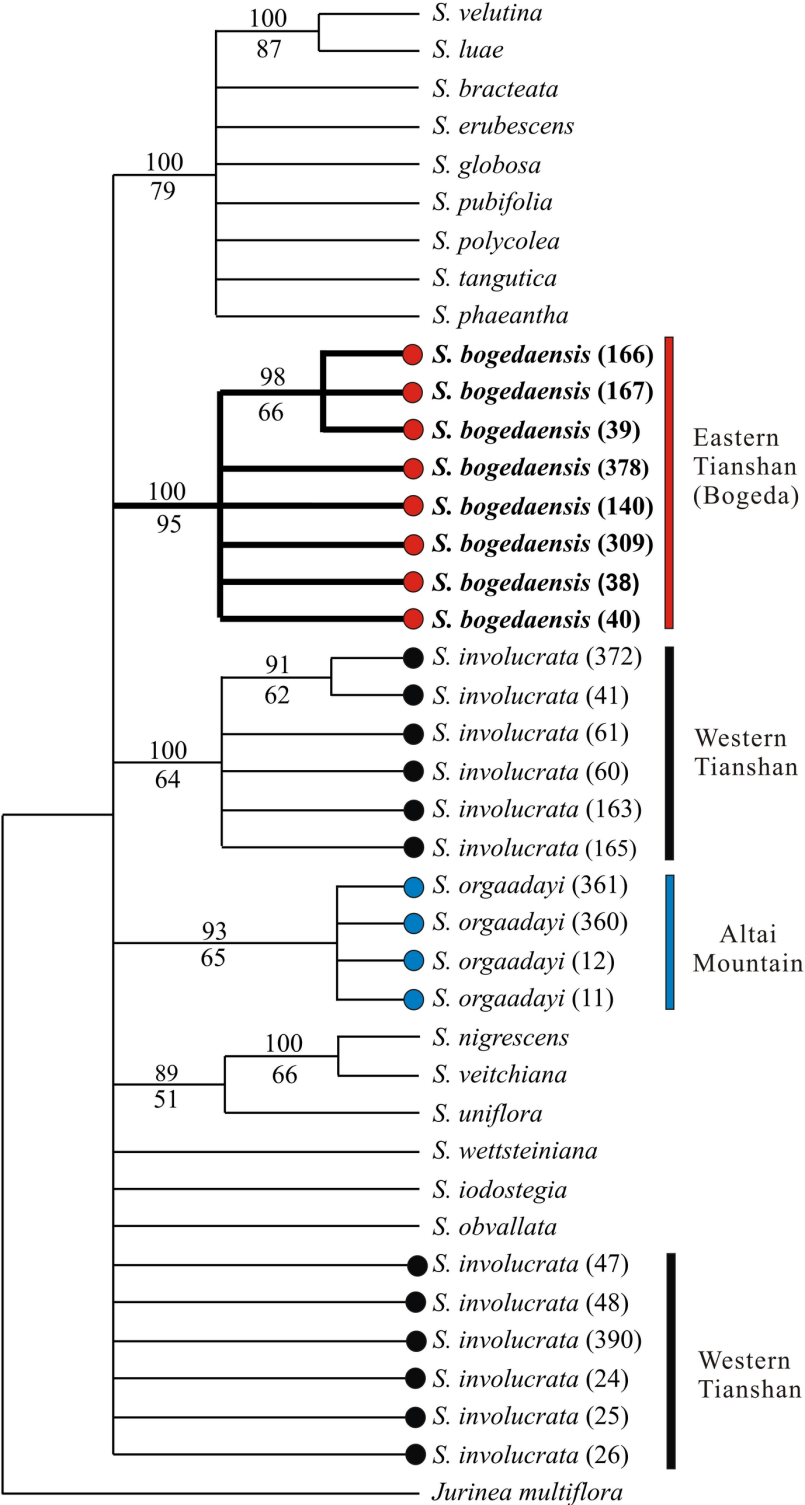
The 50% majority rule consensus tree derived from Bayesian analysis of the combined plastid dataset. Posterior probabilities (PPs) and bootstrap percentages (BPs) are indicated above and below the branches, respectively.

The aligned combined plastid (*psb*A*-trn*H, *trn*K, and *mat*K) matrix contained 1551 characters, 49 of which were variable and 14 were phylogenetically informative. Similar to the results based on the ITS sequences, the pairwise distances of the combined cp loci between *S*. *bogedaensis* and *S*. *involucrata* or *S*. *orgaadayi* were both 0.3%, which was the smallest among the pairwise distances between *S*. *bogedaensis* and the other species (Table 4). The trees obtained by MP and BI were mainly congruent and the latter is shown in Fig 6. Both *S*. *bogedaensis* (PP = 93; BP = 65%) and *S*. *orgaadayi* (PP = 93%; BP = 65%) were resolved as monophyletic. However, those from *S*. *involucrata* failed to form a monophyletic group. Moreover, the three species did not form a monophyletic clade (Fig 6).

The combination of ITS and plastid matrix obtained similar tree from BI and MP, and the former was shown in S1 Fig. The topology is highly similar to that from ITS, but support for a few clades, including that containing *S*. *involucrate*, is a little higher than that from ITS (PP = 100%; BP = 96%).

## Discussion

As one of the four *Saussurea* subgenera, subg. *Amphilaena* is defined mainly by colored uppermost leaves or bracts surrounding the synflorescence [16,17,35]. A recent study indicated that this character might have been derived more than once and that this subgenus might be polyphyletic [36,37], but no new infrageneric system has been proposed for *Saussurea* or subg. *Amphilaena*. Thus, we tentatively ascribed the new species to subg. *Amphilaena*. In subg. *Amphilaena*, *S*. *involucrata* and *S*. *orgaadayi* were identified as similar species to the new species because of a morphological combination unique to these species, i.e, the cream-yellow bracts that aggregated below the florescence and the hollow stem at least 1.5 cm in diameter near base. Their morphological affinity was also supported by our molecular analyses. Thus, the genetic distances between the new species and *S*. *involucrata* were 0.98% based on ITS and 0.3% for cp, where were the smallest among the new species and the other sampled in-group species. Moreover, the three species resolved into a well-supported clade in the ITS phylogeny (Fig 5).

The three species are closely related in terms of both their morphology and molecular level characteristics, but they also have significant differences. First, six morphological differences were identified among the three species based on multiple individuals from at least two populations for each species. In particular, the shapes of the involucre and the abaxial indumenta are distinct in each species, whereas the other characters differ in at least two species. Second, all three species were resolved into three monophyletic clades, which were well supported and they corresponded to the morphological divisions in the ITS phylogeny. Third, all three species are geographically isolated. Thus, the Tianshan Mountains and Altai Mountains are separated by the Junggar Basin. In the Tianshan Mountains, the western and eastern parts are separated by Chaiwopu Basin (Fig 1). Both basins might be sufficiently large to impede or reduce gene flow among these regions, especially for plants that inhabit high altitude regions. Accordingly, we propose that these species might be derived from a common ancestor, but they may have differentiated after reaching their current range due to restricted gene flow.

In plants, closely related species often share the same common chemical components [38–40]. Thus, it is reasonable to hypothesize that the new species may have similar medicinal value to *S*. *involucrata* because of their very high similarity and recent differentiation. However, the population of the new species might be rather small. We found this species in two localities, which were both located in restricted areas immediately below peaks that were covered with snow all the year around, where we only found six mature individuals and 50 immature individuals. This harsh environment might at least partly explain their rarity. Thus, we suggest that exploitation of this new species should be subject to strict protection.

## Taxonomic treatment

***Saussurea bogedaensis*** Yu J. Wang & J. Chen **sp. nov.** [urn:lsid:ipni.org:names: 77180814–1] (Figs 2, 3, 4C and 4F) **Type:** China. Xinjiang: Qitai Country, Banjiegou Town, Bogeda Mountain, 43.45321°N, 89.55213°E, 3471 m, July 22, 2016, *WYJ201607018* (holotype, LZU).

**Diagnosis**. Similar to *S*. *involucrata* or *S*. *orgaadayi* but differs in terms of acuminate and densely pubescent phyllaries in middle-upper part.

**Description.** Herbs 15–50 cm tall, perennial. Caudex stout, unbranched, densely covered with fibrous remains of petioles. Stem solitary, 1.5–3 cm in diam., erect, simple. Rosette and stem leaves petiolate; leaf blade narrowly ovate, elliptic, or obovate, 15–20 × 3–5 cm, both surfaces green and glandular hairy, base decurrent, margin denticulate to serrulate, apex obtuse. Uppermost stem leaves sessile, ovate to elliptic, 5.5–12 × 3.5–6.5 cm, membranous, stellate surrounding synflorescence, both surfaces pale yellow. Capitula 15–30 in a hemispheric synflorescence, 8–15 cm in diam., sessile or shortly pedunculate. Involucre broadly campanulate, 1–2.5 cm in diam. Phyllaries in four or five rows, subulate, light brown with dark margin, densely pubescent on middle-upper part, apex acuminate; outer phyllaries 25–30 × 2.5–4 mm; middle and inner phyllaries 18–23 × 1.5–3 mm. Receptacle papillose; papillae 0.5–1 mm. Corolla purple, 1.3–1.8 cm, tube 7–9 mm, limb 6–9 mm, lobes 3–5 mm. Achene straw-colored with blackish spots, cylindrical 4.8–6.7 mm. Pappus dirty white; outer bristles 0.5–3 mm; inner bristles 0.8–1.5 cm.

**Distribution.** The species is currently known only from two localities in Bogeda Mountain located in Qitai, Xinjiang, China.

**Conservation Status**. we discovered only six individuals in blossom, all without mature seeds, and no more than 50 immature ones in cliffs near the snowline of the Bogeda Mountain. We estimated the species comprise less than 500 individuals in the light of its restrict distribution. Due to its highly resembling to *S*. *involucrata*, there is risk of harvest by herb-digger and/or native shepherd. We propose that the location should be recognized as critical habitat and the species listed as ‘‘Critically Endangered” according to the IUCN red list categories and criteria [41].

## Supporting information

S1 Fig BI of all the sequences in combination. The 50% majority rule consensus tree derived from Bayes inference of the combined sequences of nuclear ITS and all the plastid loci. Posterior probabilities and bootstrap percentages are indicated above and below the branches, respectively.
(TIF)
