# Supplementary figures and images for: New Saussurea (Asteraceae) species from Bogeda Mountain, eastern Tianshan, China, and inference of its evolutionary history and medical usage
Source: PLoS One. 2018 Jul 18;13(7):e0199416. doi: 10.1371/journal.pone.0199416 (PMC6051572; doi:10.1371/journal.pone.0199416)

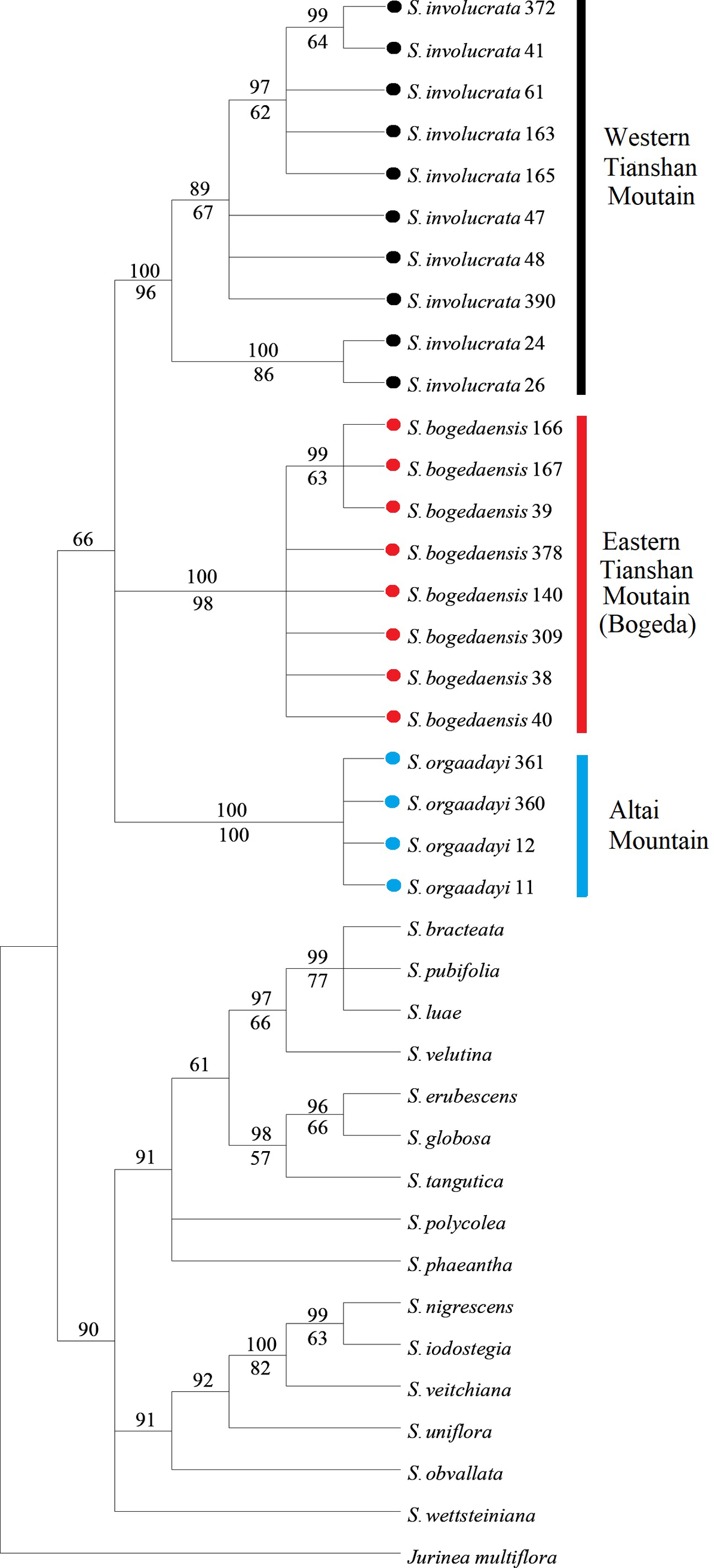

Supplement: S1 Fig — The 50% majority rule consensus tree derived from Bayes inference of the combined sequences of nuclear ITS and all the plastid loci. Posterior probabilities and bootstrap percentages are indicated above and below the branches, respectively. (TIF) [file pone.0199416.s001.tif]
